# Antero‐Lateral Subthalamic Nucleus Theta Stimulation Improves Verbal Fluency in Parkinson's Disease

**DOI:** 10.1002/mds.30185

**Published:** 2025-04-02

**Authors:** Hannah Schoenwald, Bahne H. Bahners, Silja Kannenberg, Till A. Dembek, Michael T. Barbe, Dafina Sylaj, Anja Spiewok, Saskia Elben, Tomke Muettel, Jan Vesper, Philipp Slotty, Alfons Schnitzler, Stefan J. Groiss

**Affiliations:** ^1^ Institute of Clinical Neuroscience and Medical Psychology, Medical Faculty and University Hospital Düsseldorf, Heinrich Heine University Düsseldorf Düsseldorf Germany; ^2^ Department of Neurology, Center for Movement Disorders and Neuromodulation, Medical Faculty and University Hospital Düsseldorf Heinrich Heine University Düsseldorf Düsseldorf Germany; ^3^ Department of Neurology, Faculty of Medicine University of Cologne Cologne Germany; ^4^ Department of Neurology Hospital Leverkusen Leverkusen Germany; ^5^ Department of Functional Neurosurgery and Stereotaxy, Department of Neurosurgery, Medical Faculty and University Hospital Düsseldorf Heinrich Heine University Düsseldorf Düsseldorf Germany; ^6^ Clinic for Orthopaedics and Trauma Surgery, Functional Neurosurgery and Neuromodulation, Helios St. Josefhospital Uerdingen Krefeld Germany; ^7^ Neurocenter Düsseldorf Germany

**Keywords:** deep brain stimulation, neurocognitive side effects, low‐frequency stimulation, verbal fluency sweet spot, left hemisphere

## Abstract

**Objective:**

Low‐frequency deep brain stimulation (DBS) of the subthalamic nucleus (STN) has been associated with positive effects on verbal fluency (VF) in patients with Parkinson's disease. This prospective study investigates stimulation direction‐dependent and site‐specific effects of theta frequency DBS on VF.

**Methods:**

In a double‐blind, cross‐over design (n = 20), we tested VF during left subthalamic theta stimulation (stimulation‐off, omnidirectional, and threedirectional stimulation conditions). DBS electrode localization and electric field calculations were performed (n = 18). Probabilistic sweet spot mapping identified voxels with significant change in VF.

**Results:**

Best directional stimulation improved VF performance significantly compared with the stimulation‐off and omnidirectional stimulation condition. This effect followed a medial‐to‐anterolateral gradient with higher VF improvement observed on the border between the motor and associative subparts of the STN.

**Conclusion:**

We provide first proof‐of‐principle evidence that directional theta frequency DBS improves VF, possibly related to stimulation of the anterolateral STN. © 2025 The Author(s). *Movement Disorders* published by Wiley Periodicals LLC on behalf of International Parkinson and Movement Disorder Society.

High‐frequency bilateral deep brain stimulation (DBS) of the subthalamic nucleus (STN) has proven effective to treat cardinal motor symptoms in patients with Parkinson's disease (PwP) with some reports of mild neuropsychological side effects.^1^, ^2^, ^3^, ^4^ One frequently reported neuropsychological side effect of high‐frequency STN‐DBS is the deterioration of verbal fluency (VF).^5^, ^6^, ^7^, ^8^, ^9^ A reduction of stimulation frequency from 130 Hz to 10 Hz resulted in better VF performance and a non‐significant trend of better VF compared with DBS‐Off.^10^ Another study suggested an improvement of VF performance under theta stimulation of the left dorsal STN.^11^

Reports on stimulation site‐specific effects within the left STN vary.^5^, ^9^, ^11^, ^12^ Most of the mentioned studies on VF effects of DBS are based on small patient cohorts and are often limited by insufficient blinding and a lack of control conditions.^7^, ^9^, ^13^ Previously, with conventional, ring‐shaped DBS electrodes the investigation of directed current administration to STN targets and its effect on VF was not possible.^5^, ^14^, ^15^, ^16^, ^17^ Therefore, the aim of this study was to probe the effects of directional left‐hemisphere low‐frequency (LF) STN‐DBS on phonemic VF in PwP in a prospective, single‐center, randomized controlled design. We hypothesized that directional LF‐STN‐DBS allows for a stimulation site‐specific improvement of VF performance.

## 1 Methods

Twenty PwP with directional STN‐DBS were recruited for this double‐blind study. Patient demographics, characteristics and chronic stimulation parameters are detailed in Table S1 and S2. Patients underwent five different phonemic VF tests (Regensburger Wortflüssigkeitstest, RWT) under different left STN stimulation conditions (off‐stimulation [*DBS‐off*], omnidirectional stimulation [*oDBS*], and stimulation of each of the three directional contacts [*dDBS*]), using contact level 3,^11^ while *off‐*medication, in a randomized order. Stimulation settings were set to 6 Hz, 60 μs, 3 mA for oDBS and 2 mA for dDBS, to compensate for the total electrical energy delivered (TEED) under directional stimulation.^11^, ^12^ The study design is summarized in Figure S1.

Statistical analyses were performed using the lme4 and emmeans packages in R (Version 4.2.3). A linear mixed effects model (LME) with stimulation condition as fixed effect, patient as random effect, and VF performance as the dependent variable was fitted. To assess the effect of stimulation direction, dDBS contacts were categorized into anterior, medial, lateral, and posterior based on the determined contact orientation degree. We tested the effect of contact orientation and contact degree on VF performance separately using LME with patient as random effect. If applicable, post‐hoc paired *t*‐tests were performed to compare the respective categorical variable levels. *P*‐values < 0.05 were considered significant for all tests.

We used Lead‐DBS to localize DBS electrodes and to perform the sweet spot mapping analysis.^18^, ^19^ Two patients had to be excluded from the Lead‐DBS analysis due to missing postoperative imaging (n = 18). Although Abbott electrodes have a small marker size, directional electrode orientations were correctly determined in 15/18 electrodes using DioDe,^20^ when compared with X‐ray images (see Table S3). We estimated electric fields for each stimulation setting^21^ and then identified voxels with significantly above or below average change in VF. A nonparametric permutation statistic was used to control for errors due to multiple comparisons and within‐subject effects.

## 2 Results

### 2.1 Effects of Low‐Frequency DBS on VF Performance

**FIG. 1:**
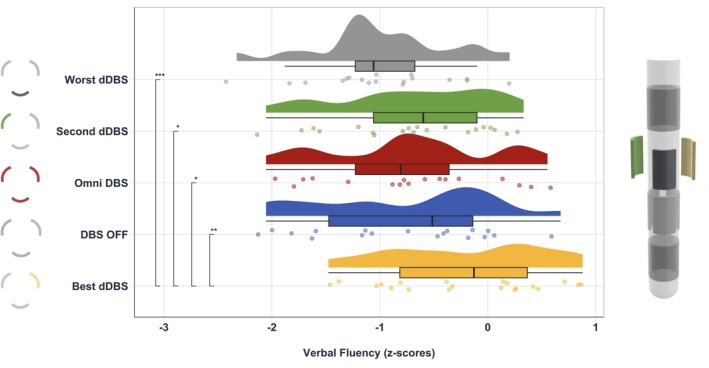
Verbal fluency mean z‐scores (Regensburger Wortflüssigkeitstest, RWT) across stimulation conditions. Asterisks represent the post‐hoc test results after multiple comparison correction using Tukey's method between conditions (*P < 0.05, **P < 0.01, ***P < 0.001). Left column shows exemplary contact orientations. dDBS, directional deep brain stimulation. [Color figure can be viewed at wileyonlinelibrary.com]

Regarding the effect of stimulation condition on VF performance, results indicated a significant main effect [*F* (4, 69.238) = 9.354, *P* < 0.001]. Post‐hoc tests (Table S4) revealed a significant difference between best‐dDBS and each of the other conditions (Figs 1 and S2): *best‐dDBS* versus *oDBS* [*t*(69.5) = 3.34, *P* = 0.001], *best‐dDBS* versus *DBS‐off* [*t*(69.4) = 4.034, *P* = 0.011], *best‐dDBS* versus *second‐best‐dDBS* [*t*(69) = 3.385, *P* = 0.01], and *best‐dDBS* versus *worst‐dDBS* [*t*(69.3) = 5.939, *P* = 0.001]. Neither *contact orientation* nor *contact degree* had a significant main effect on VF performance in the respective LME.

### 2.2 TEED, Condition Order, and VF Task Letter

The comparison between the TEED with the oDBS and all dDBS settings showed a significantly higher TEED for oDBS in a *t*‐test for related samples (*P*_*best*_ < 0.001; *P*_*second‐best*_ < 0.001; *P*_*worst*_ = 0.0012). When labeling directional stimulation settings based on the anatomical contact orientations (lateral, medial, anterior, posterior, and omnidirectional) and fitting the linear mixed model using VF improvement as dependent variable and contact orientation and TEED as fixed effects, we neither observed a significant main effect of contact orientation nor TEED on VF improvement [*TEED*: *F* (1, 46.000) = 2.5093, *P* = 0.1200, contact orientation: *F* (4, 40.689) = 0.4525, *P* = 0.7699]. Post‐hoc results are detailed in Table S5. Further results are reported in the supplementary material and Figure S5.

No main effects of *condition order* or *VF task letter* on VF performance were found [*F*_*order*_ (1, 69.864) = 1.463, *P* = 0.231; *F*_*letter*_ (4, 67.212) = 1.54, *P* = 0.201].

### 2.3 Probabilistic Stimulation Mapping

**FIG. 2:**
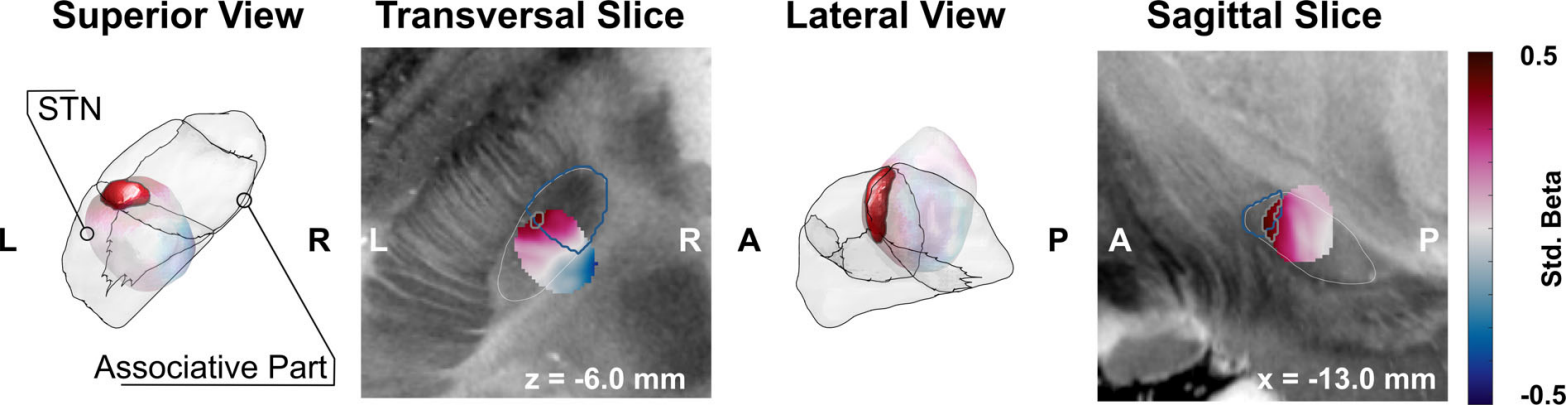
Probabilistic stimulation mapping of the z‐scored verbal fluency (VF) improvement in three‐ and two‐dimensional views. VF improvement is shown in the weighted mean‐effect image (outliers excluded) and scaled according to standardized regression coefficient (see color bar on right). The cluster of voxels with significant above‐average improvement is highlighted, with non‐significant voxels in transparent colors. The subthalamic nucleus (STN) according to the DISTAL atlas is outlined in grey. L, left; R, right; A, anterior; P, posterior. [Color figure can be viewed at wileyonlinelibrary.com]

After pooling all investigated stimulation fields for voxel‐wise analysis, the resulting weighted mean image showed a clear medial‐to‐anterolateral gradient with higher VF improvement observed toward the (atlas defined)^22^ border to the associative subpart of the STN and anterolateral to it (Fig. 2). Voxel‐wise statistical analysis revealed a cluster of voxels associated with better‐than‐average VF improvement centered on the dorsolateral border of the associative subpart of the STN, but this cluster failed to reach statistical significance during non‐parametric permutation testing (rank 689/1000; *P* = 0.311). Further analyses are detailed in the supplementary material and Figures S3, S4 and S6.

## 3 Discussion

The aim of this study was to investigate the site‐specific effects of left STN theta frequency DBS on VF performance in PwP. In comparison with the DBS‐off and oDBS conditions, the best‐dDBS setting elicited significantly better VF outcomes (Fig. 1). Voxelwise analysis suggested that stimulation at the border between the motor and associative subparts of the STN might be related to a higher‐than‐average VF improvement, but so far statistical evidence to confirm this assumption is lacking.

### 3.1 Directional Theta Frequency DBS Improves VF Performance

There was a significant difference between VF performance in the best‐dDBS and oDBS condition as well as between the best‐dDBS and DBS‐off condition. Previous studies with smaller sample sizes were only able to find positive trends for LF‐DBS on VF^10^ or a VF improvement but only compared with the off‐condition.^11^ In fact, omnidirectional LF‐DBS did not result in a significant difference in VF improvement compared with the stimulation‐off condition in our study, which could also hint towards a site‐specific VF effect and could explain why earlier work did not find a strong effect of LF‐DBS on VF performance.^5^ Our findings provide evidence of VF performance improvement through directional theta DBS in a double‐blind, randomized controlled study design.

### 3.2 Spatial Specificity of Theta DBS Effects on VF Improvement in the Associative STN

Neither contact orientation nor contact degree explained a significant amount of variance in VF improvement in the respective models. If we expect a spatially specific effect within the STN, this should not only be reflected by the contact orientation but also – maybe more importantly – the anatomical location of the DBS lead itself in relation to the STN. When translating the analysis to anatomical space, we found a medial‐to‐anterolateral gradient of VF improvement. The results of our LME show a contact‐dependent relationship between distance to sweet spot and VF, with anterior contact stimulation showing the strongest relationship without significant differences between contact orientations in post‐hoc tests. Ultimately, our sweet spot analysis addresses the effect of anatomical location on VF in a more elaborate way than our LME and is better suited to resolving all the anatomical information relevant for DBS‐related improvements that the variables included in our linear mixed models fail to capture. However, a cluster of voxels with significantly better‐than‐average VF within the associative subpart of the STN failed to reach overall significance in the non‐parametric permutation analysis.

Individual theta‐frequency dDBS was shown to significantly improve VF.^11^ With newly available sensing‐enabled neurostimulators, the adjustment towards the individual theta‐frequency peak for VF modulation is feasible and could be used to further tune the modulatory effects on VF in a personalized manner.^23^ These developments will help gain a better understanding of low‐frequency oscillations in the STN and might eventually facilitate treatment options through more specific targeting and personalized stimulation paradigms.

### 3.3 Limitations

With a sample size of 20 patients the observed effects might not be generalizable to larger cohorts of PwP, even though the effect sizes of post‐hoc tests were relatively high. Our study protocol was based on several assumptions from previous research and the effect of other DBS contacts or stimulation frequencies was not accounted for in this study. Also, we did not record motor scores for the tested settings.

Given the variable anatomical locations and orientations of DBS leads across patients, the categorization of directional contacts is challenging. We decided to rank dDBS contacts based on VF performance. To a certain extent, this inflates the effect on VF improvement, especially comparing dDBS contacts. However, the main focus of our analysis is the difference between the *DBS‐off* and *dDBS* conditions. Our findings with ranked dDBS contacts were further supported by the anatomical findings, even though they did not translate to the two‐dimensional variables of contact orientation and degree. Finally, none of the experimental circumstances such as condition order or VF task letter had an effect on VF performance that would have had to be accounted for in our LME models (see Supplementary Material; Data S1). TEED was higher in the *oDBS* than the *dDBS* condition, suggesting an effective compensation of contact impedance differences by the adjusted stimulation amplitude for directional contacts.

Probabilistic mapping results were most likely impacted by the limited amount of data. While voxel‐wise mapping revealed a clear spatial gradient and a cluster of voxels associated with better‐than‐average VF improvement, the lack of statistical confirmation during non‐parametric permutation analysis suggests insufficient power of these findings.

### 3.4 Outlook

Future studies are needed to replicate our results within larger cohorts exploring a larger number of stimulation settings. Regarding the orientation and placement of each patient's electrode, a multi‐frequency or interleaving stimulation of both high‐ and low‐frequency DBS could be tested to investigate whether theta‐frequency DBS can counteract negative side effects of high‐frequency DBS, allowing for an individually optimized treatment for PwP in the future.

## 4 Conclusions

We provide the first proof‐of‐principle evidence that directional theta‐frequency STN‐DBS improves VF performance compared with omnidirectional and DBS‐off conditions. Our results support the notion of stimulation site‐specific effects of dDBS within the STN on VF performance and may potentially provide new opportunities to counteract negative effects of high‐frequency STN‐DBS on VF.

## Author Roles

(1) Research Project: A. Conception, B. Organization, C. Execution; (2) Statistical Analysis: A. Design, B. Execution, C. Review and Critique; (3) Manuscript Preparation: A. Writing of the First Draft, B. Review and Critique. H.S.: Conception; Organization and Execution of research project; Design and execution of statistical analysis; Lead DBS analysis; Manuscript preparation (first draft and reviews). B.H.B.: Statistical analysis (execution); Lead DBS analysis; manuscript review and critique. S.K.: Statistical analysis (execution); Lead DBS analysis; manuscript review and critique. T.A.D.: Lead DBS analysis; probabilistic stimulation mapping; manuscript review. M.T.B.: Manuscript review. D.S.: Execution of research project. A.Sp.: Execution of research project. S.E.: Research project conception; manuscript review. T.M.: Execution of research project. J.V.: Manuscript review. P.S.: Manuscript review. A.Sch: Organization of research project, manuscript review. S.J.G.: Conception and organization of research project, manuscript review.

## Financial Disclosures

H.S.: None. B.H.B.: Support from the Prof. Dr. Klaus Thiemann Foundation (Parkinson Fellowship 2022). S.K.: None. T.A.D.: Speaker honoraria: Movement Disorder Society and Medtronic. Travel support from Boston Scientific. M.T.B.: Research funding: Felgenhauer‐Stiftung, Forschungspool Klinische Studien and Köln Fortune (University of Cologne), Horizon 2020 (Gondola), Medtronic (ODIS, OPEL, BeAble), Boston Scientific. Consultancies: IQWIG, Medtronic, Esteve, Boston Scientific, AbbVie. Honoraria: Medtronic, Boston Scientific, Abbott (formerly St. Jude), FomF, derCampus, GE Medical, UCB, Bial, Apothekerverband Köln e.V., BDN, Ever Pharma, Esteve. D.S.: None. A.Sp.: None. S.E.: None. T.M.: None. J.V.: None. P.S.: None. A.Sch.: Grants: Deutsche Forschungsgemeinschaft. Consultancies/Honoraria: Abbott, AbbVie, Alexion, BSH Medical Communication, Kyowa Kirin, Novartis, Zambon. S.J.G.: Consultancies: AbbVie, Bial. Honoraria: Abbott, Boston Scientific, Inomed.

## Supporting information


**Data S1.** Supporting information.

## Data Availability

The data that support the findings of this study are available from the corresponding authors upon reasonable request.
