# Supplementary material for: Antero‐Lateral Subthalamic Nucleus Theta Stimulation Improves Verbal Fluency in Parkinson's Disease
Source: Mov Disord. 2025 Apr 2;40(6):1195–9. doi: 10.1002/mds.30185 (PMC12160961; doi:10.1002/mds.30185)
Supplement: Supplementary file 1 — Data S1. Supporting information. [file MDS-40-1195-s001.docx]

**Supplementary Material**

**Supplementary Methods**

***Participants***

The medical research ethics committee of the Heinrich-Heine University of Duesseldorf gave ethical approval for this work (study number: 2021-1505), which was conducted in accordance with the Declaration of Helsinki. Each participant provided written informed consent prior to study participation.

20 PwP (15 male, mean age: 68.3 years ± 8.16) were recruited between January and August 2022 at the Center for Movement Disorders and Neuromodulation at the University Hospital Düsseldorf. Patient demographics are summarized in Supplementary Table 1a. Sample size was based on proof of principle, considering previous smaller sample sizes (n=9)^1^ and unknown effect sizes. All participants had a clinical diagnosis of Parkinson’s disease and had been implanted with directional DBS electrodes into the STN bilaterally prior to this study. Responsiveness to levodopa was assessed prior to the implantation and all patients were rated on part III of the Unified Parkinson’s disease Rating Scale (MDS-UPDRS-III) in separate check-ups, showing a significant DBS effect across the cohort (Med OFF, Stim OFF vs. Med OFF Stim ON, *P* ≤ .001). Individual demographic measures are detailed in Supplementary Table 1b. Chronic DBS settings and levodopa equivalent daily doses (LEDD) are reported in Supplementary Table 2. Patients were excluded from the study if prior neuropsychological testing showed any indication of dementia in the Mattis dementia rating scale (MDRS)^2^ and/or the Montreal Cognitive Assessment (MoCA)^3^, (cut-offs: MDRS: 130/144, MoCA: 19/30).

***Procedure***

All 20 patients were tested in the *off-*medication state after overnight withdrawal of dopaminergic medication for at least 12 hours. To rule out cross-stimulation effects, VF performance was rated at least 20 minutes after each DBS setting change. The order of stimulation settings was randomized for each patient. The examiner and patient were blinded to the stimulation condition during the VF tasks, which was changed by an independent assistant. In each of the five stimulation conditions, patients performed one neuropsychological VF task from the “Regensburger Wortflüssigkeitstest” (RWT), testing phonemic VF with different initial letters (“P”,”B”,”M”,”K”,”S”). The order of letters was randomized for each patient to prevent sequence effects and the examiner was blinded to the order (see Supplementary Fig. 1). Patients were asked to produce as many corresponding words as possible within one minute, while avoiding repetition of the same word and names. For each VF-task the total number of correct words was recorded and later transformed into z-scores. Differences in z-scores were used for statistical analysis.

***Stimulation conditions***

VF-performance (n=20) was compared between the five stimulation conditions: Off-stimulation (*DBS-off*), omnidirectional stimulation (*oDBS*) and stimulation of each of the three directional contacts (*dDBS*). Based on a previous study reporting a VF stimulation site spanning the whole vertical axis of the associative STN and an improvement of VF with dorsal low frequency DBS only, we used contact level 3, i.e. the uppermost level with directional contacts, across patients.^1^ The stimulation frequency was set to 6 Hz, the pulse width to 60 µs.^1^ Omnidirectional stimulation was applied with a stimulation intensity of 3 mA to the left STN, while directional stimulation was applied with 2 mA. We chose different stimulation intensities to compensate for the total electrical energy delivered (TEED),^4^ i.e. to avoid higher stimulation intensities at directional contacts that might cause stronger effects of dDBS on VF. We further calculated the TEED using the Koss formular for each of the stimulation contacts^5^ and tested differences as described in the Statistics section. DBS of the right hemisphere remained turned off during testing. The order of conditions was randomized and both the examiner and patient were blinded to the conditions (see Supplementary Fig. 1). The chronic DBS settings at the time of the study are reported in Supplementary Table 2.

***Lead localization***

Two patients had to be excluded from the Lead-DBS analysis, due to missing postoperative imaging (n = 18). To analyze the role of stimulation direction and investigate a stimulation sweetspot for VF-improvement, we localized DBS electrodes with the advanced processing pipeline^6^ in Lead-DBS.^7^ Postoperative CT or MRI images of all participants were linearly coregistered to preoperative MRI images, using the advanced normalization tools.^8^ Results of coregistration were refined if necessary, followed by a brain shift correction step. In order to estimate a precise multispectral normalization to ICBM 2009b NLIN asymmetric (“MNI”) space^9^ all preoperative volumes were used. The ANTs SyN Diffeomorphic Mapping^10^ with the preset ”effective: low variance default + subcortical refinement” was used. Alternatively a multispectral implementation of the unified segmentation approach^11^ withing Statistical Parametric Mapping software (SPM12, fil.ion.ucl.ac.uk/spm)^12^ was applied, for patients where this strategy failed. Through these preset-methods, the STN was segmented with precision comparable to manual expert segmentations.^13^ This step was followed by automatic pre-reconstruction of DBS contacts with the phantom-validated and fully-automated PaCER method^14^ or the TRAC/CORE approach, including manual refinement if necessary. To reconstruct the segmented leads we used the Directional Orientation Detection (DiODe) algorithm.^15^ Except for one patient, all participants had Abbott directional leads (see Supplementary Table 1b). The DiODe algorithm was only validated for Boston Scientific directional leads, so far.^16^ Thus, DiODe results for Abbott leads were critically reviewed for plausibility. Besides ensuring that the DiODe results match the X-ray orientations, we visually inspected the lead with the manual orientation tool implemented in LeadDBS. In case DiODe failed to detect an orientation matching the X-ray images or in case there was postoperative MRI only, we manually adjusted the lead orientation to an angle of 0° (anterior), 90° (medial), 180° (posterior) and -90° (lateral) for the left STN-electrode orientation based on the X-ray. For details see Supplementary Table 3. The DISTAL atlas^13^ defines atlas segmentations and with the Lead group toolbox^17^ group visualizations were performed.

***Statistical analyses***

Statistical analyses were performed using the lme4 and emmeans packages in R (Version 4.2.3). First, we performed a non-parametrical Spearman’s correlation between education in years and MoCA as well as MDRS to assess a potential effect of education in years on screening tests used for patient selection in this study. VF task results were transformed into z-scores based on the number of words generated per minute.^18^ Outliers in VF data were detected using the 99^th^ percentile and rejected for further analyses, given the strong deviation from the mean when visualizing the data. Data were evaluated for normality using the Shapiro-Wilk-test. For the directional stimulation conditions, outcomes were ranked as best, second best and worst VF performance (conditions: “*Best-dDBS*”, “*second-best-dDBS”* and “*worst-dDBS*”). A linear mixed effects model (LME) with stimulation condition as fixed effect, patient as random effect and VF performance as the dependent variable was fitted, one including and one excluding outliers. Next, to assess the effect of stimulation direction we categorized the directional stimulation contacts into four categories (anterior, medial, lateral and posterior) based on the contact orientation degree determined as described above. We tested the effect of contact orientation and contact degree on VF performance separately using LME with patient as random effect. To rule out sequence effects or effects of specific letters from the RWT task on VF performance, we computed separate LME with condition order or VF task letter as fixed effects respectively.

If applicable, post-hoc paired t-tests were performed to compare the respective categorical variable levels. *P*-values were corrected for multiple comparisons using Tukey’s method. A paired t-test was performed for comparison between the TEED for omnidirectional and all directional stimulation settings. *P*-values < 0.05 were considered significant for all tests.

***Probabilistic Stimulation Mapping***

Two patients had to be excluded from the probabilistic stimulation mapping, due to missing postoperative imaging (n = 18). For each stimulation setting, electric fields were estimated using FastField assuming a homogenous tissue conductivity of 0.1 S/m.^19^ Electric fields were then transformed to activation probabilities ranging between 0 and 1 using a sigmoid function based on previously published thresholds (see supplementary figure 4).^20,21^

All stimulation fields were then nonlinearly transformed to the common MNI template for voxel-wise analysis. All voxels not receiving a cumulative activation probability of 15 from different stimulation fields were discarded from further analysis. For the remaining voxels, a weighted mean-effect image was created by assigning each voxel the mean VF outcome of the stimulation fields affecting this voxel, weighted by the respective activation probabilities. Then, for each voxel, its respective weighted mean was tested against the overall average VF improvement using a weighted linear-regression analysis – analogous to a two-sample t-test but including weights for each observation according to its respective activation probability. This analysis can then identify voxels with significant above or below average change in VF. Clusters of significant voxels were identified and evaluated using the sum of all t-values of the voxels within a cluster. As in other DBS mapping analyses^22–25^, each stimulation setting was used as an independent observation and no per‑patient random effects can be accounted for in such an analysis. To address this issue as well as the increased risk of false positives in voxel‑wise statistics due to multiple comparisons, we employed a post‑hoc nonparametric permutation statistic. VF outcomes of one lead were randomly redistributed to stimulation fields from another lead – while maintaining the within-subject “connectedness” of the outcomes and thus accounting for per‑patient random effects^25^. This type of permutation was repeated n = 1000 times and the sum of all t‑values of the largest cluster of significant voxels within each permutation was calculated. To confirm the statistical validity of clusters identified in our original data, the sum of t-values from the original cluster (summary statistic) can then be ranked against the summary statistics from permuted dataset. We assumed significance of our original findings only if the summary statistic ranked higher than 950/1000 - in line with a significance level of 0.05. Additionally, the electric fields for the chronic stimulation settings (see Supplementary Table 2) were calculated and the volume where at least one third of clinically used stimulations (n=6) overlapped was computed to compare it with the VF voxels (see Supplementary Figure 3).

**Supplementary Results**

Patient characteristics are summarized in Supplementary Table 1a&b. Two patients had to be excluded from the probabilistic stimulation mapping analysis and the model on VF effects of contact orientation and degree, due to missing postoperative imaging (n = 18). None of the participants reported any side effects of stimulation changes and were reset to their chronic DBS settings after the study.

***Effects of low frequency DBS on VF-performance***

Post-hoc test results: *best-dDBS* vs *oDBS* (*t*(69.5) = 3.34, *P* = .001, *Cohen’s d* = 1.11, 95% CI [0.42, 1.8]), *best-dDBS* vs *DBS-off* (*t*(69.4) = 4.034, *P* = .011, *Cohen’s* *d* = 1.32, 95% CI [0.63, 2.00]), *best-dDBS* vs. *second-best-dDBS* (*t*(69) = 3.385, *P* = .01, *Cohen’s d* = 1.07, 95% CI [0.41, 1.7]), *best-dDBS* vs. *worst-dDBS* (*t*(69.3) = 5.939, *P* ≤ .001, *Cohen’s d* = 1.94, 95% CI [1.22, 2.65]).

Without outlier exclusion we observe the same main effects in the linear mixed effects models. The post-hoc differences between best directional contact and DBS-Off as well as omnidirectional stimulation also remain. Only the difference between best and second-best directional DBS contact does not reach statistical significance, when keeping the outliers. We now report both results - with and without outlier exclusion in Supplementary Table 4.

We fit another linear mixed effect model using anatomical location (defined as the distance to a published motor sweet spot) and contact orientation as fixed effects, patient as random effect and verbal fluency as the dependent variable. We use the distance of each of the electrode contacts to a published motor sweet spot^26^ to have a reference point within the STN enabling us to define the anatomical location of each electrode in relation to that spot. First, when only including distance to the sweet spot into the model we find a significant effect of that variable on verbal fluency (*F* (1, 52.914) = 6.7223, *P* = 0.01228). Second, when fitting the full model, we find a main effect of contact orientation and a significant interaction effect between anatomical location and contact orientation (Contact Orientation: *F* (4, 40.214) = 3.1368, *P* = 0.02459, Anatomical Location: *F* (1, 37.923) = 3.2170, *P* = 0.08086, Interaction: *F* (4, 40.570) = 2.6067, *P* = 0.04972). When examining the interaction plot (supplementary figure 5), we observe a contact-dependent relationship between distance to sweet spot and verbal fluency, with anterior contact stimulation showing the strongest relationship. However, the post-hoc tests did not reveal significant differences between contact orientations (Supplementary Table 5).

Neither *contact-orientation* nor *contact-degree* had a significant main effect on VF-performance in the respective LME (*contact orientation*: *F*(3, 33.084) = 0.034, *P* = .992; *contact degree*: *F*(1, 34.833) = 0.001, *P* = .997).

***TEED, condition order and VF task letter***

The comparison between the TEED with the omnidirectional (*M* = 3.15, *SD* = 0.74) and all directional experimental stimulation settings (*M_best_* = 2.23, *SD_best_* = 0.725; *M_second-best_* = 2.24, *SD_second-best_* = 0.85; *M_worst_* = 2.34, *SD_worst_* = 1.09) showed a significantly higher TEED for omnidirectional stimulation in a t-test for related samples (*P_best_* < .001; *P_second-best_* < .001; *P_worst_* = .0012). There was no significant difference in TEED between best and second-best directional stimulation (*P* = .95) or between best and worst directional stimulation (*P* = .68). No main effects of *condition* *order* or *VF task* *letter* on VF performance were found (*order*: *F*(1, 69.864) = 1.463, *P* = .231; *letter*: *F*(4, 67.212) = 1.54, *P* = .201).

***Probabilistic Stimulation Mapping***

Voxel-wise statistical analysis revealed a cluster of voxels associated with higher-than-average VF improvement centered on the dorso-lateral border of the associative subpart of the STN (Supplementary Fig. 3). No other clusters were identified. The identified cluster failed to reach overall significance after nonparametric permutation testing (Supplementary Fig. 6). The comparison of chronic DBS volume overlap with the VF stimulation site showed a dorso-medial stimulation site outside of and posterior to the VF site (Supplementary Fig. 3).

**Supplementary Tables**

**Supplementary Table 1a: Mean** **demographic measures of participants.**

|  | **N** | **Age** | **Education** | **Disease duration** | **DBS duration** | **DBS System** | **Mean Stimulation intensity (mA)** | **MDRS points^c^** | **MoCA points^c^** |
| --- | --- | --- | --- | --- | --- | --- | --- | --- | --- |
|  |  | **(in years)** | **(in years)** | **(in years)** | **(in months)** |  | **of left electrode** |  |  |
| **Participants** | 20 | 64.9^a^ (8.16)^b^ | 14.35^a^ (2.53)^b^ | 12.4^a^ (7.06)^b^ | 20.57^a^ (21.32)^b^ | 19 Abbott Infinity | 1.68^a^ (1.06)^b^ | 140.31^a^/144 (3.40)^b^ | 24.83^a^/30 (2.87)^b^ |
|  |  |  |  |  |  | 1 Boston Scientific |  |  |  |
| **Female** | 5 | 63.4^a^ (11.54)^b^ | 13.2^a^ (0.90)^b^ | 13.6^a^ (6.02)^b^ | 18.6^a^ (16.11)^b^ | 4 Abbott Infinity | 1.71^a^ (1.11)^b^ | 139.5^a^/144 (1.91)^b^ | 24.25^a^/30 (2.87)^b^ |
|  |  |  |  |  |  | 1 Boston Scientific |  |  |  |
| **Male** | 15 | 65.4^a^ (7.40)^b^ | 14.73^a^ (2.86)^b^ | 12^a^ (7.53)^b^ | 19.39^a^ (23.14)^b^ | 15 Abbott Infinity | 1.67^a^ (0.87)^b^ | 140.53^a^/144 (3.71)^b^ | 25^a^/30 (2.96)^b^ |

^a^mean

^b^standard deviation

**Supplementary Table 1b: Demographic measures of all participants individually.**

| **Patient** | **Age^a^** | **Gender** | **Education^a^** | **Disease** | **DBS** | **DBS** | **MDRS** | **MoCA** |
| --- | --- | --- | --- | --- | --- | --- | --- | --- |
| **ID** |  |  |  | **Duration^a^** | **Duration^b^** | **System** | **score** | **score** |
| 1 | 53 | m | 20 | 16 | 3 | Abbott Infinity | 141/144 | 29/30 |
| 2 | 72 | w | 13 | 10 | 2 | Abbott Infinity | 139/144 | 28/30 |
| 3 | 64 | m | 13 | 4 | 0.25 | Abbott Infinity | 143/144 | 20/30 |
| 4 | 61 | m | 15 | 18 | 58 | Abbott Infinity | 142/144 | 23/30 |
| 5 | 73 | m | 11 | 13 | 0.22 | Abbott Infinity | 137/144 | 17/30 |
| 6 | 67 | m | 13 | 5 | 0.22 | Abbott Infinity | 140/144 | 27/30 |
| 7 | 69 | m | 13 | 8 | 1.6 | Abbott Infinity | 136/144 | 22/30 |
| 8 | 59 | w | 13.5 | 11 | 2 | Abbott Infinity | 137/144 | 24/30 |
| 9 | 71 | m | 18 | 7 | 1 | Abbott Infinity | 144/144 | 30/30 |
| 10 | 65 | w | 13 | 20 | 21 | Abbott Infinity | 141/144 | 24/30 |
| 11 | 68 | m | 12 | 9 | 37.36 | Abbott Infinity | 142/144 | 23/30 |
| 12 | 75 | w | 12 | 20 | 32 | Abbott Infinity | 141/144 | 21/30 |
| 13 | 73 | m | 13 | 17 | 65.56 | Abbott Infinity | 144/144 | missing |
| 14 | 49 | m | 14 | 4 | 5.03 | Abbott Infinity | 142/144 | 26/30 |
| 15 | 74 | m | 13 | 12 | 12 | Abbott Infinity | 143/144 | 25/30 |
| 16 | 58 | m | 15 | 11 | 51 | Abbott Infinity | 142/144 | 22/30 |
| 17 | 65 | m | 19 | 12 | 2 | Abbott Infinity | 144/144 | 28/30 |
| 18 | 69 | m | 19 | 10 | 29 | Abbott Infinity | 131/144 | 23/30 |
| 19 | 46 | m | 14,5 | 7 | 36 | Boston Scientific^c^ | missing | missing |
| 20 | 66 | m | 19 | 34 | 26.67 | Abbott Infinity | 137/144 | 25/30 |

^a^ in years

^b^ in months

^c^ Boston Scientific Cartesia

**Supplementary Table 2: Chronic stimulation parameters & LEDD**

| Patient | Left hemisphere | Right hemisphere | Frequency (Hz) | Pulse width (µs) | Stimulation intensity  (mA) | LEDD |
| --- | --- | --- | --- | --- | --- | --- |
| ID | contact | contact | Left/right | Left & right | Left/right |  |
| 1 | 3ABC-/ G+ | 11ABC-/ G+ | 130/130 | 60 | 0.5/0.5 | 830 |
| 2 | 2ABC/ G+ | 10ABC-/ G+ | 130/130 | 60 | 1/1 | 910 |
| 3 | 2ABC/ G+ | 10ABC-/ G+ | 130/130 | 60 | 0.5/1.3 | 840 |
| 4 | 2B-/ G+ | 10C-/ G+ | 130/130 | 60 | 1.6/1.8 | 100 |
| 5 | -2ABC/ G+ | -10ABC-/ G+ | 130/130 | 60 | 0.5/0.5 | 510 |
| 6 | 2C- / G+ | 10A-/ G+ | 130/130 | 60 | 0.4/1.2 | 1380 |
| 7 | 2ABC/ G+ | 11ABC-/ G+ | 130/130 | 60 | 1/1 | 2260 |
| 8 | 2ABC-/G+ | 10ABC-/ G+ | 130/130 | 60 | 1.2/1.2 | 630 |
| 9 | -4/ G+ | 12-/ G+ | 130/130 | 60 | 3/2.5 | 180 |
| 10 | 10A-/ G+ | 2A-/ G+ | 130/130 | 60 | 1.45/1.45 | 530 |
| 11 | 1-/ G+ | 10B-/ G+ | 130/130 | 60 | 2.9/1.4 | 0 |
| 12 | 3C-/ G+ | 11C-/ G+ | 160/160 | 60 | 3.2/1.2 | 470 |
| 13 | 2B-/ G+ | 10B-/ G+ | 130/130 | 60 | 2.3/3 | 1320 |
| 14 | -2ABC/ G+ | -11ABC-/ G+ | 130/130 | 60 | 1.5/3.6 | 1030 |
| 15 | 3ABC-/ G+ | 11ABC-/ G+ | 130/130 | 60 | 2.3/1.2 | 670 |
| 16 | 11A-/ G+ | 4-/ G+ | 130/130 | 60 | 1.65/1.6 | 760 |
| 17 | 2 ABC/ G+ | 10ABC-/ G+ | 130/130 | 60 | 0.7/0.8 | 1590 |
| 18 | 4-/ G+ | 11ABC-/ G+ | 130/130 | 60 | 3.2/2.7 | 1130 |
| 19 | 2,3,4-/ G+ | 2,3,4-/ G+ | 132/159 | 60 | 4/5 | 400 |
| 20 | 10 ABC- / G+ | 2ABC-/ G+ | 125/125 | 60 | 0.8/1.2 | 240 |

**Supplementary Table 3: Eletrode rotation in X-ray and Lead-DBS reconstruction.**

| **Patient-ID** | **X-ray** | **DioDe** | **angle (DioDe)** | **corrected** |
| --- | --- | --- | --- | --- |
| 1 | *no X-ray* | Medial | -108,2937619 |  |
| 2 | medial | Medial | -79,09860962 |  |
| 3 | medial | Medial | -97,41451848 |  |
| 4 | lateral | Lateral | 87,74912918 |  |
| 5 | medial | anteromedial | -38,34589714 |  |
| 6 | anterior | anterior | 20,29739214 |  |
| 7 | posterolateral | Lateral | 92,35395091 |  |
| 8 | anteromedial | *no reconstruction* | *no reconstruction* |  |
| 9 | anterolateral | anterolateral | 27,7522104 |  |
| 10 | posterolateral | posteromedial | -134,9859308 | 135 |
| 11 | posterior | posterolateral | 117,41536 |  |
| 12 | anterolateral | anterior | 15,89063442 |  |
| 13 | posteromedial | anterior | -12,27686969 | -81 |
| 14 | medial | posterolateral | 135 | -90 |
| 15 | anteromedial | anteromedial | -25,70524258 | -68 |
| 16 | posteromedial | posterior | -176,315105 |  |
| 17 | posterior | posterior | -159,4172514 |  |
| 18 | posterolateral | posteromedial | -135 | 135 |
| 19 | anterolateral | *no reconstruction* | *no reconstruction* |  |
| 20 | posterolateral | posteromedial | -135 | 135 |

**Supplementary Table 4: Post hoc analysis of stimulation conditions.**

1. **With outlier exclusion**

| contrast | estimate | SE | df | t.ratio | *P*.value |
| --- | --- | --- | --- | --- | --- |
| best dDBS - off | 0.6119 | 0.152 | 69.4 | 4.034 | 0.0013** |
| best dDBS - omnidirectional | 0.5160 | 0.154 | 69.5 | 3.340 | 0.0114* |
| best dDBS - second best dDBS | 0.4960 | 0.147 | 69.0 | 3.385 | 0.0100* |
| best dDBS - worst dDBS | 0.8994 | 0.151 | 69.3 | 5.939 | <.0001*** |
| off - omnidirectional | -0.0959 | 0.157 | 69.1 | -0.610 | 0.9730 |
| off - second best dDBS | -0.1159 | 0.152 | 69.4 | -0.764 | 0.9400 |
| off - worst dDBS | 0.2875 | 0.157 | 69.7 | 1.834 | 0.3625 |
| omnidirectional - second best dDBS | -0.0200 | 0.154 | 69.5 | -0.129 | 0.9999 |
| omnidirectional - worst dDBS | 0.3834 | 0.160 | 69.9 | 2.403 | 0.1267 |
| second best dDBS - worst dDBS | 0.4034 | 0.151 | 69.3 | 2.664 | 0.0700* |

1. **Without outlier exclusion**

| contrast | estimate | SE | df | t.ratio | p.value |
| --- | --- | --- | --- | --- | --- |
| best dDBS - off | 0.9284 | 0.239 | 75.0 | 3.879 | 0.0020** |
| best dDBS - omnidirectional | 0.8557 | 0.243 | 75.2 | 3.519 | 0.0064** |
| best dDBS - second best dDBS | 0.4960 | 0.239 | 75.0 | 2.072 | 0.2429 |
| best dDBS - worst dDBS | 1.1160 | 0.239 | 75.0 | 4.663 | 0.0001** |
| off - omnidirectional | -0.0727 | 0.243 | 75.2 | -0.299 | 0.9982 |
| off - second best dDBS | -0.4324 | 0.239 | 75.0 | -1.807 | 0.3775 |
| off - worst dDBS | 0.1876 | 0.239 | 75.0 | 0.784 | 0.9345 |
| omnidirectional - second best dDBS | -0.3597 | 0.243 | 75.2 | -1.479 | 0.5789 |
| omnidirectional - worst dDBS | 0.2603 | 0.243 | 75.2 | 1.071 | 0.8210 |
| second best dDBS - worst dDBS | 0.6200 | 0.239 | 75.0 | 2.590 | 0.0824 |

**P* < 0.05.

***P* < 0.01

****P* < 0.001

**Supplementary Table 5: Post-hoc Test for linear mixed effect model.**

Anatomical location (defined as the distance to a published motor sweet spot) and contact orientation used as fixed effects, patient as random effect and verbal fluency as the dependent variable.

contrast estimate SE df t.ratio p.value

Anterior location3.35- Lateral location3.35 0.2585 0.214 38.7 1.210 0.7459

Anterior location3.35- Medial location 3.35 0.1895 0.214 37.1 0.887 0.8898

Anterior location 3.35- Omnidirectional location 3.35 0.3694 0.183 35.8 2.021 0.2772

Anterior location 3.35- Posterior location 3.35 0.2714 0.222 36.7 1.222 0.7389

Lateral location 3.35- Medial location 3.35 -0.0690 0.226 37.4 -0.305 0.9980

Lateral location 3.35- Omnidirectional location 3.35 0.1109 0.201 37.7 0.553 0.9809

Lateral location 3.35- Posterior location 3.35 0.0129 0.236 37.9 0.055 1.0000

Medial location 3.35- Omnidirectional location 3.35 0.1799 0.208 37.0 0.866 0.9073

Medial location 3.35- Posterior location 3.35 0.0819 0.241 37.2 0.340 0.9970

Omnidirectional location 3.35- Posterior location 3.35 -0.0980 0.214 36.5 -0.459 0.9905

**Supplementary figures**

**Supplementary figure 1: Study design.**

**
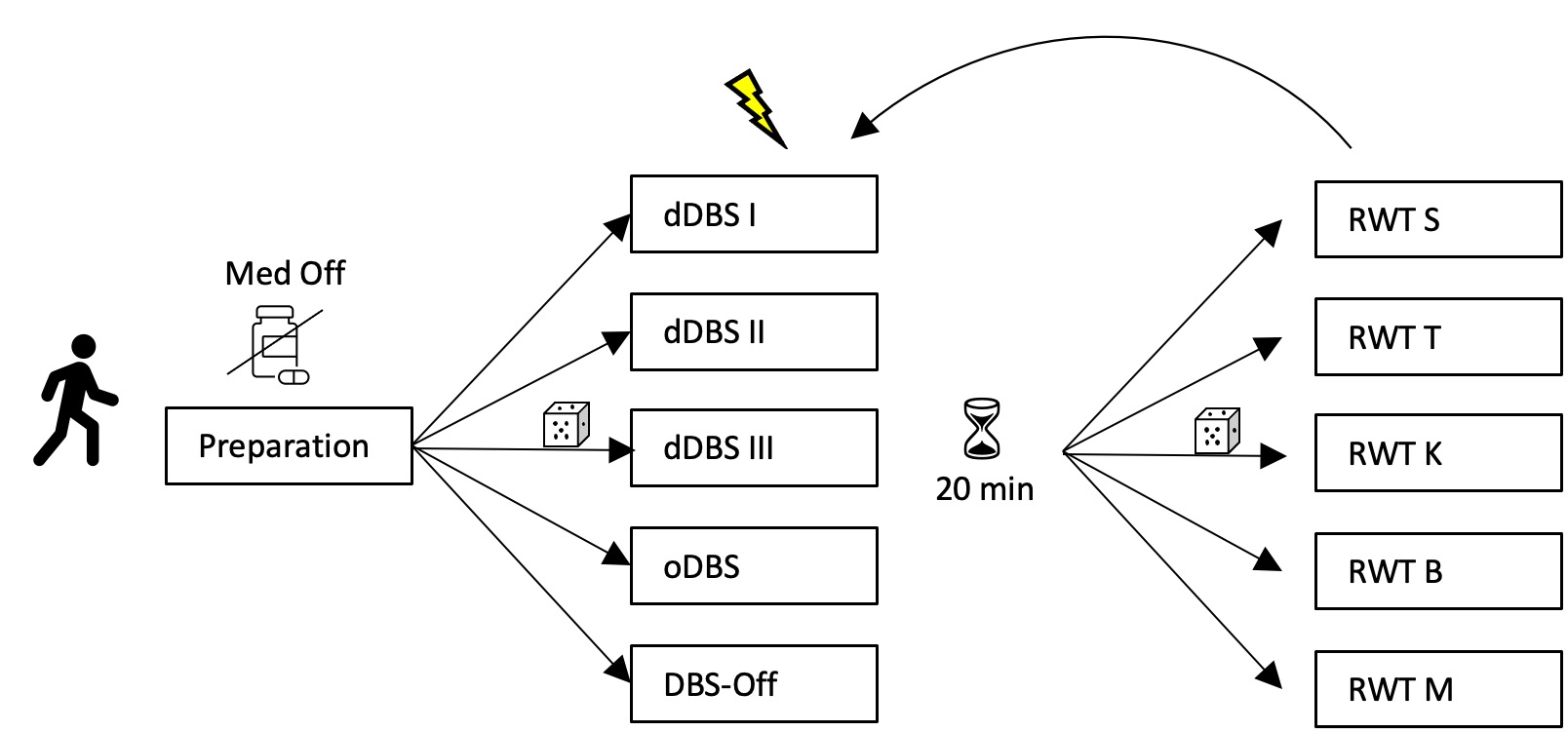
**

**Supplementary figure 2: Boxplots for VF performance.**

Results shown for anterior, lateral, medial and posterior directional DBS, DBS-Off and omnidirectional stimulation.


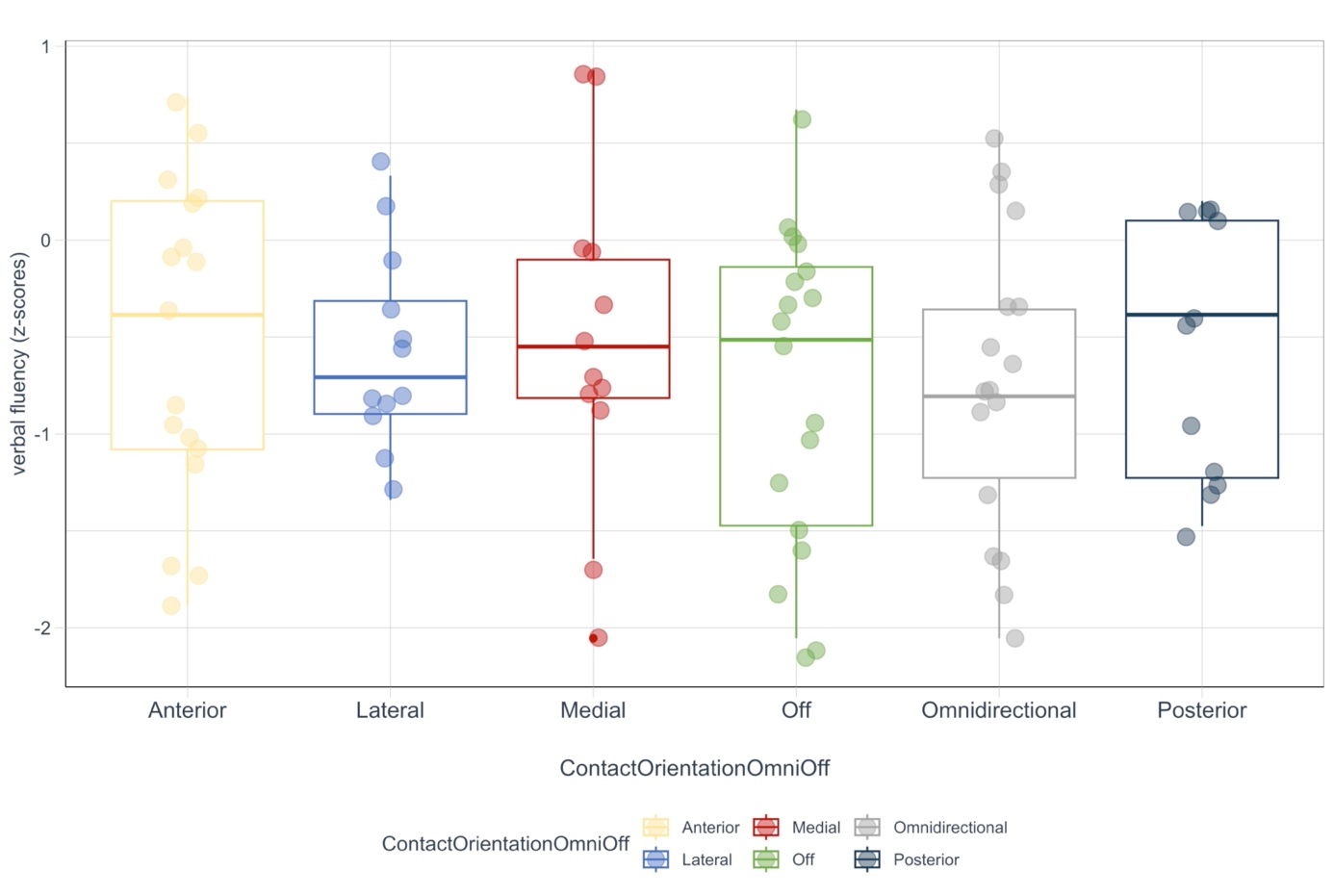


**Supplementary figure 3**: **Normalized locations of all investigated leads.**

Calculation without outliers (upper row) and volume where at least one third of clinically used stimulations (n=6) overlapped (lower row, green) in relation to the sweetspot for VF-improvement (yellow), the STN according to the DISTAL atlas (outlined in grey).


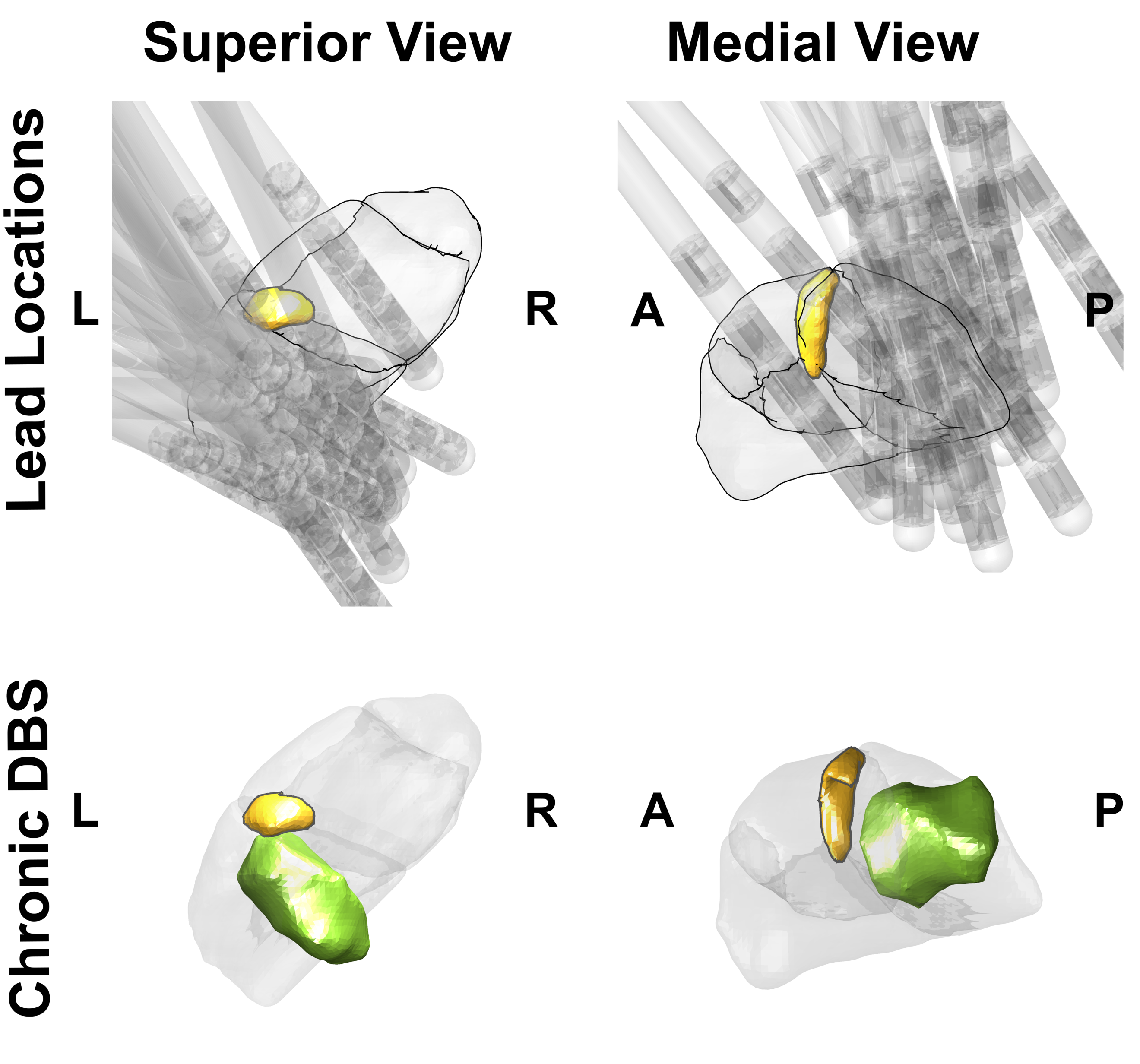


**Supplementary figure 4: Activation probability.**

Based on the electric field thresholds published by Astrom et al.^21^, we derived a sigmoidal activation function. The function ranged from 0 % activation probability to 100 %. The function was fitted in a way that a 5 % activation probability was reached at an electric field of 0.061 V/mm (lowest published estimate for axons with a diameter of 7.5µm) while a 95 % activation probability was reached at an electric field of 0.351 V/mm (highest published estimate for axons with a diameter of 2.5µm). The resulting function meant that a 50 % activation probability was reached at an electric field of 0.206 V/mm - close to the commonly used threshold of 0.2 V/mm in many publications using binarized VTAs.


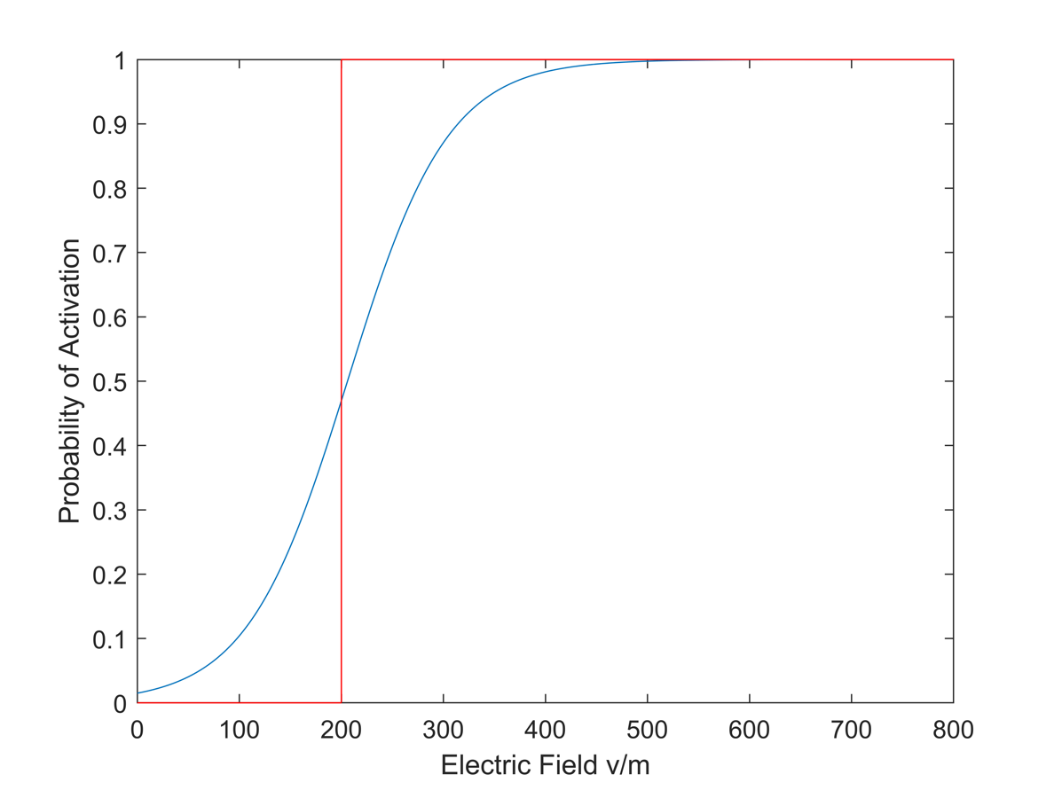


**Supplementary figure 5: Contact-dependent relationship between distance to sweet spot and verbal fluency.**

The anterior contact stimulation shows the strongest relationship. Post-hoc tests did not reveal significant differences between contact orientations.


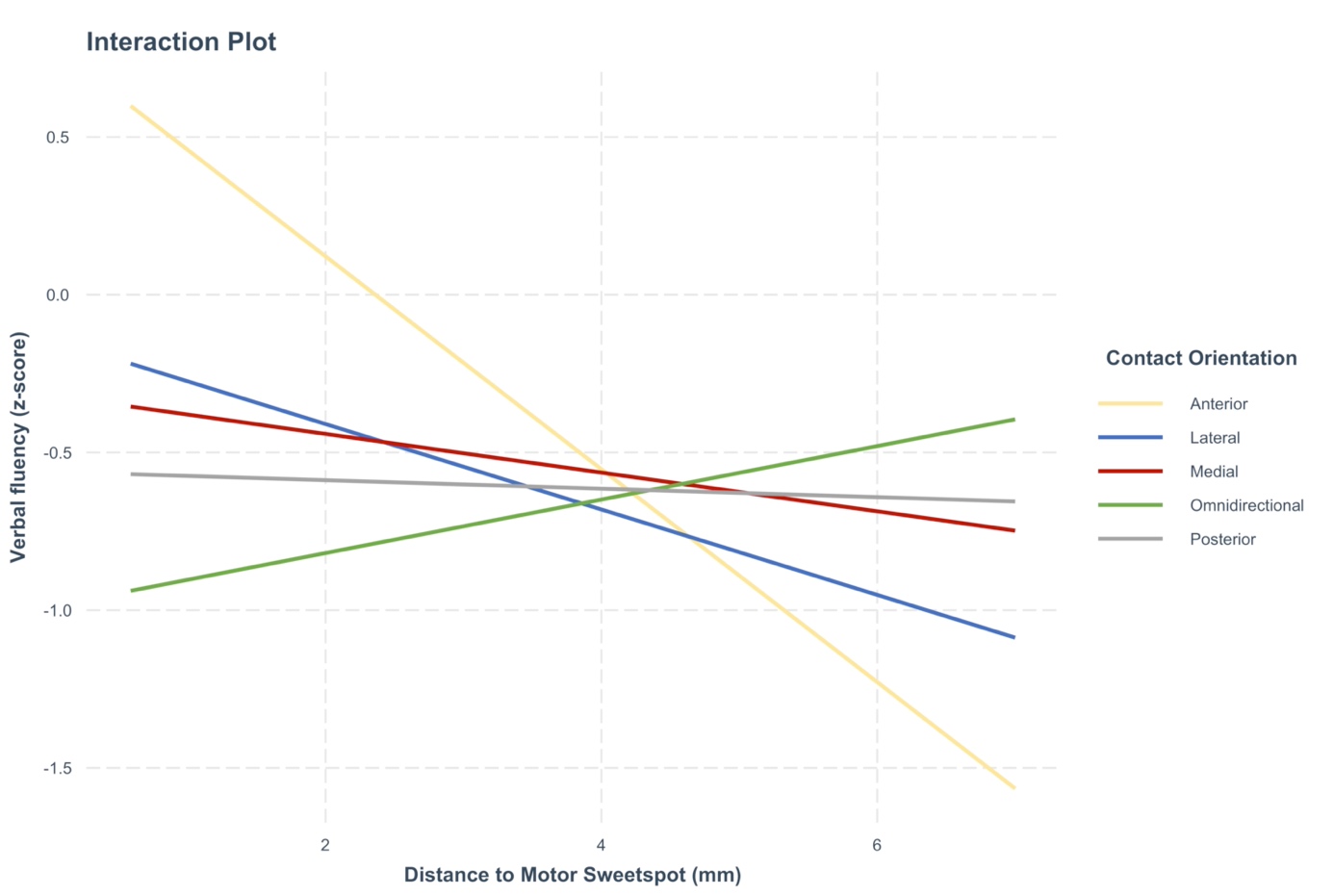


**Supplementary figure 6: Nonparametric permutation statistic.**

The summary t-statistic of the identified cluster for better-than-average VF improvement (red line) is shown against the distribution of summary t-statistics from n = 1000 permutations. Being ranked 689/1000, the cluster failed to reach overall statistical significance (p = 0.311).

*
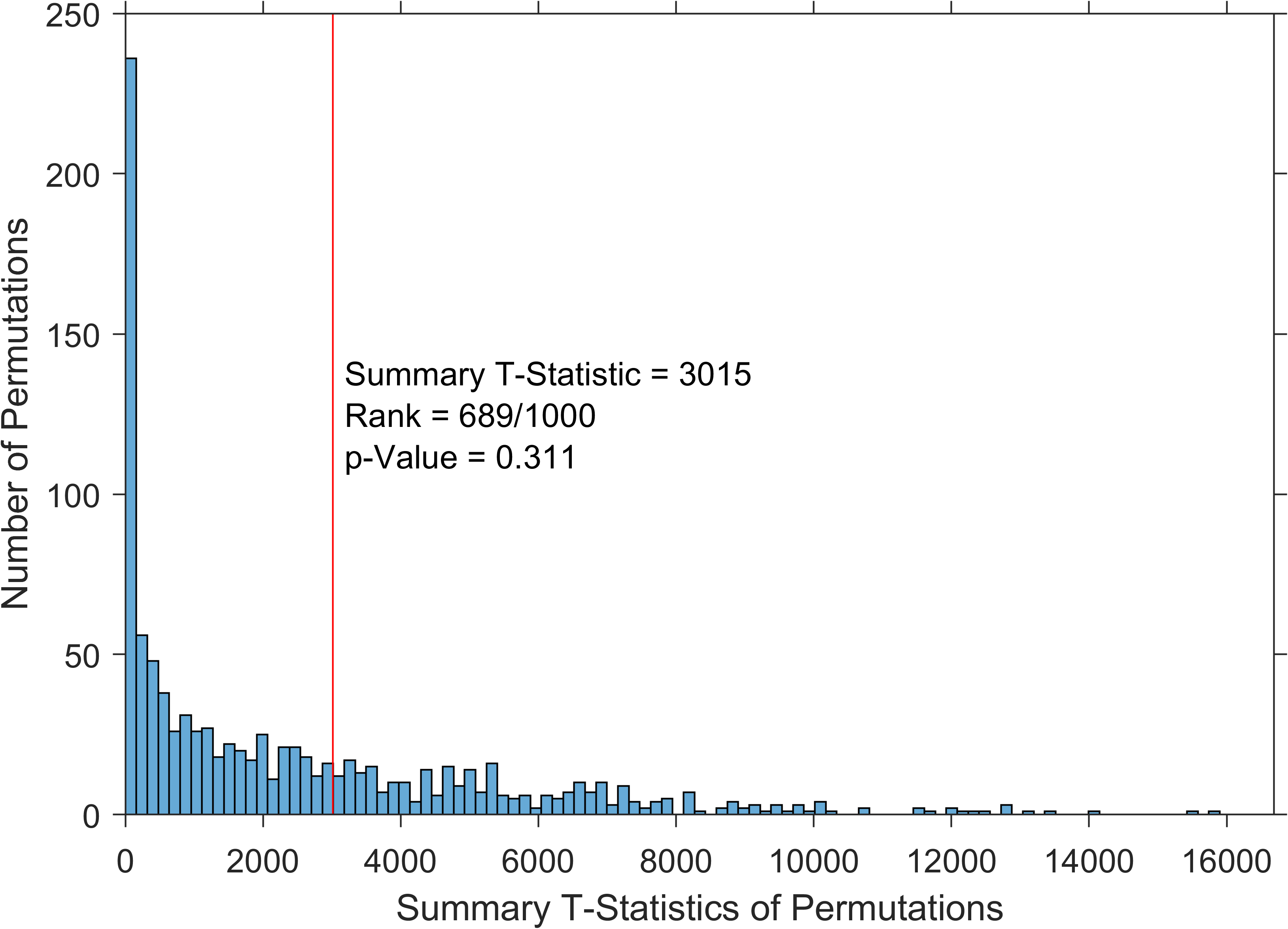
*

References

1. Lee DJ, Drummond NM, Saha U, et al. Acute low frequency dorsal subthalamic nucleus stimulation improves verbal fluency in Parkinson’s disease. *Brain Stimulation*. 2021;14(4):754-760. doi:10.1016/j.brs.2021.04.016

2. Mattis S. Dementia Rating Scale (DRS). Published online 1988.

3. Nasreddine ZS, Phillips NA, Bédirian V, et al. The Montreal Cognitive Assessment, MoCA: A Brief Screening Tool For Mild Cognitive Impairment: MoCA: A brief screening tool for MCI. *Journal of the American Geriatrics Society*. 2005;53(4):695-699. doi:10.1111/j.1532-5415.2005.53221.x

4. Moro E, Esselink RJA, Xie J, Hommel M, Benabid AL, Pollak P. The impact on Parkinson’s disease of electrical parameter settings in STN stimulation. *Neurology*. 2002;59(5):706-713. doi:10.1212/WNL.59.5.706

5. Koss AM, Alterman RL, Tagliati M, Shils JL. Calculating total electrical energy delivered by deep brain stimulation systems. *Ann Neurol*. 2005;58(1):168-168. doi:10.1002/ana.20525

6. Horn A, Li N, Dembek TA, et al. Lead-DBS v2: Towards a comprehensive pipeline for deep brain stimulation imaging. *NeuroImage*. (184):293-316. doi:https://doi.org/10.1016/j.neuroimage.2018.08.068

7. Horn A, Kühn AA. Lead-DBS: A toolbox for deep brain stimulation electrode localizations and visualizations. *NeuroImage*. 2015;107:127-135. doi:10.1016/j.neuroimage.2014.12.002

8. Avants BB, Tustison NJ, Song G, Cook PA, Klein A, Gee JC. A reproducible evaluation of ANTs similarity metric performance in brain image registration. *NeuroImage*. 2011;54(3):2033-2044. doi:10.1016/j.neuroimage.2010.09.025

9. Fonov V, Evans AC, Botteron K, Almli CR, McKinstry RC, Collins DL. Unbiased average age-appropriate atlases for pediatric studies. *NeuroImage*. 2011;54(1):313-327. doi:10.1016/j.neuroimage.2010.07.033

10. Avants B, Epstein C, Grossman M, Gee J. Symmetric diffeomorphic image registration with cross-correlation: Evaluating automated labeling of elderly and neurodegenerative brain. *Medical Image Analysis*. 2008;12(1):26-41. doi:10.1016/j.media.2007.06.004

11. Ashburner J, Friston KJ. Unified segmentation. *NeuroImage*. 2005;26(3):839-851. doi:10.1016/j.neuroimage.2005.02.018

12. Friston KJ, Holmes AP, Worsley KJ, Poline JP, Frith CD, Frackowiak RSJ. Statistical parametric maps in functional imaging: A general linear approach. *Hum Brain Mapp*. 1994;2(4):189-210. doi:10.1002/hbm.460020402

13. Ewert S, Plettig P, Li N, et al. Toward defining deep brain stimulation targets in MNI space: A subcortical atlas based on multimodal MRI, histology and structural connectivity. *NeuroImage*. 2018;170:271-282. doi:10.1016/j.neuroimage.2017.05.015

14. Husch A, V. Petersen M, Gemmar P, Goncalves J, Hertel F. PaCER - A fully automated method for electrode trajectory and contact reconstruction in deep brain stimulation. *NeuroImage: Clinical*. 2018;17:80-89. doi:10.1016/j.nicl.2017.10.004

15. Dembek TA, Hoevels M, Hellerbach A, et al. Directional DBS leads show large deviations from their intended implantation orientation. *Parkinsonism & Related Disorders*. 2019;67:117-121. doi:10.1016/j.parkreldis.2019.08.017

16. Sitz A, Hoevels M, Hellerbach A, et al. Determining the orientation angle of directional leads for deep brain stimulation using computed tomography and digital x‐ray imaging: A phantom study. *Medical Physics*. 2017;44(9):4463-4473. doi:10.1002/mp.12424

17. Treu S, Strange B, Oxenford S, et al. Deep brain stimulation: Imaging on a group level. *NeuroImage*. 2020;219:117018. doi:10.1016/j.neuroimage.2020.117018

18. Aschenbrenner S, Tucha O, Lange KW. *Regensburger Wortflüssigkeits-Test: RWT.* Hogrefe, Verlag für Psychologie; 2000.

19. Baniasadi M, Proverbio D, Gonçalves J, Hertel F, Husch A. FastField: An open-source toolbox for efficient approximation of deep brain stimulation electric fields. *NeuroImage*. 2020;223:117330. doi:10.1016/j.neuroimage.2020.117330

20. Jergas H, Petry-Schmelzer JN, Hannemann J, et al. One side effect – two networks? Lateral and postero-medial stimulation spreads induce dysarthria in subthalamic deep brain stimulation for Parkinson’s Disease. *medRxiv*. Published online January 1, 2023:2023.04.26.23289100. doi:10.1101/2023.04.26.23289100

21. Astrom M, Diczfalusy E, Martens H, Wardell K. Relationship between Neural Activation and Electric Field Distribution during Deep Brain Stimulation. *IEEE Trans Biomed Eng*. 2015;62(2):664-672. doi:10.1109/TBME.2014.2363494

22. Akram H, Sotiropoulos SN, Jbabdi S, et al. Subthalamic deep brain stimulation sweet spots and hyperdirect cortical connectivity in Parkinson’s disease. *NeuroImage*. 2017;158:332-345. doi:10.1016/j.neuroimage.2017.07.012

23. Butson CR, Cooper SE, Henderson JM, Wolgamuth B, McIntyre CC. Probabilistic analysis of activation volumes generated during deep brain stimulation. *NeuroImage*. 2011;54(3):2096-2104. doi:10.1016/j.neuroimage.2010.10.059

24. Dembek TA, Barbe MT, Åström M, et al. Probabilistic mapping of deep brain stimulation effects in essential tremor. *NeuroImage: Clinical*. 2017;13:164-173. doi:10.1016/j.nicl.2016.11.019

25. Dembek TA, Roediger J, Horn A, et al. Probabilistic sweet spots predict motor outcome for deep brain stimulation in Parkinson disease. *Annals of Neurology*. 2019;86(4):527-538. doi:10.1002/ana.25567

26. Caire F, Ranoux D, Guehl D, Burbaud P, Cuny E. A systematic review of studies on anatomical position of electrode contacts used for chronic subthalamic stimulation in Parkinson’s disease. *Acta Neurochir*. 2013;155(9):1647-1654. doi:10.1007/s00701-013-1782-1
